# 25-Year experience with adult polytraumatized patients in a European level 1 trauma center: polytrauma between 1995 and 2019. What has changed? A retrospective cohort study

**DOI:** 10.1007/s00402-022-04433-1

**Published:** 2022-04-12

**Authors:** Valerie Weihs, Stephan Frenzel, Michél Dedeyan, Florian Hruska, Kevin Staats, Stefan Hajdu, Lukas Leopold Negrin, Silke Aldrian

**Affiliations:** Department of Orthopedics and Trauma Surgery, Medical University of Vienna, Währinger Gürtel 18-20, 1090 Vienna, Austria

**Keywords:** Polytrauma, Epidemiology, Outcome, Mortality

## Abstract

**Purpose:**

To analyze the changes of the clinical characteristics, injury patterns, and mortality rates of polytraumatized patients within the past 25 years in a European Level I trauma center.

**Methods:**

953 consecutive polytraumatized patients treated at a single-level 1 trauma center between January 1995 and December 2019 were enrolled retrospectively. Polytrauma was defined as AIS ≥ 3 points in at least two different body regions. Retrospective data analysis on changes of clinical characteristics and mortality rates over time.

**Results:**

A significant increase of the average age by 2 years per year of the study could be seen with a significant increase of geriatric patients over time. No changes of the median Injury Severity Score (ISS) could be seen over time, whereas the ISS significantly decreased by patient’s year. The rates of concomitant severe traumatic brain injury (TBI) remained constant over time, and did not increase with rising age of the patients. Although, the mortality rate remained constant over time the relative risk of overall in-hospital mortality increased by 1.7% and the relative risk of late-phase mortality increased by 2.2% per patient’s year.

**Conclusion:**

The number of polytraumatized patients remained constant over the 25-year study period. Also, the mortality rates remained stable over time, although a significant increase of the average age of polytraumatized patients could be seen with stable injury severity scores. Severe TBI and age beyond 65 years remained independent prognostic factors on the late-phase survival of polytraumatized patients.

**Trial registration**: NCT04723992.

**Level of evidence**: Prognostic study, level III.

## Introduction

Although a decline in trauma-related deaths in adults can be seen over time, trauma remains one of the leading causes of death world-wide [1]. After survival of the acute phase, nearly 80% of the late-phase deaths were due to sepsis and multiple-organ failure (MOF) [2]. Within the last years, the incidence of MOF [3–6] as well as death rates due to sepsis and acute respiratory distress syndrome (ARDS) in trauma patients [4, 5, 7] showed a significant decrease. A decline of the mortality rates in multiple injured patients was found, especially between the years 1970 and 2000 [4, 5, 8] with a change of the injury patterns of trauma patients: although motor vehicle accidents remain the most common cause of injury [4, 8, 9], a decline in car crashes has been found [4]. Furthermore, an increase in the mean age of polytraumatized patients (reflecting the rapidly aging population world-wide) could be detected in recent studies [9, 10]. The incidence of traumatic brain injury (TBI) in elderly trauma patients has increased over time [11]. Recent studies suggest TBI and rising age as a strong predicting factor for survival in trauma patients [12–15]. Low-energy trauma is responsible for the late-phase mortality in about 40% of patients with traumatic brain injury as cause of death in about 26% of cases [16]. Severe TBI remains the most common cause of death in trauma patients [17–23]. Furthermore, concomitant injuries seem to have a significant effect on the mortality in patients with moderate TBI [24]. A recent study was able to demonstrate a markedly decrease in TBI-related mortality in geriatric patients over time [25].

There are few published studies reviewing the changes in trauma mechanisms, injury patterns, and outcome of polytraumatized patients over time [4, 5, 8]. The aim of this study was to integrate the above-mentioned possible prognostic factors in the analysis of a large cohort of adult polytraumatized patients regarding the possible changes over a 25-year period. Therefore, we try to answer the following questions:

- Does the mean age of polytraumatized patients increase significantly in recent years with an increase of the prevalence of geriatric polytraumatized patients over the past 25 years?
- Has the prevalence of severe TBI changed over the past 25 years?
- Can a change of trauma mechanisms with different injury patterns be seen within the past 25 years?
- Is there a decline in mortality rates of polytrauma patients over time?

## Materials and methods

In this study, 952 consecutive patients who were admitted to our level 1 trauma center with critical injuries (ISS ≥ 18 points and an Abbreviated Injury Score (AIS) ≥ 3 points in at least two body regions [26]) were enrolled retrospectively from January 1, 1995 to December 31, 2019.

### Exclusion criteria

Patients with an isolated traumatic brain injury, patients with minor injuries (AIS < 3 points or ISS < 18 points), and patients ≤ 16 years of age were excluded from this study.

Three time-dependent events for the analysis of mortality were defined: acute-phase death (death within the first 24 h or on arrival at the hospital), late-phase death (death after the first 24 h within the hospital stay), and overall death (death at any time within the hospital stay).

### Patients’ population

Nine-hundred-and-fifty-three consecutive polytraumatized patients were enrolled consecutively from January 1995 to December 2019. The data were gained from our ongoing in-hospital database of polytraumatized patients. Patients’ hospital records were reviewed and the baseline characteristics, such as gender, age, injury mechanism, and injury patterns as well as the outcomes, were reported. Geriatric patients were defined as polytraumatized patients beyond 65 years of age. Severe traumatic brain injury (TBI) was defined as AIS ≥ 3 points in the anatomical region accompanied by a Glasgow Coma Scale (GCS) ≤ 12 points. The combination of anatomical measures such as the AIS with GCS has been suggested and used before [27, 28]. Possible prognostic factors such as severe TBI, age, injury severity (ISS score), and different injury patterns were detected. The follow-up period was counted from the date of trauma to the date of the last known contact.

### Statistical analysis

Continuous variables are presented as means and standard deviations or medians and interquartile ranges depending on normal or skew distribution of the certain value. Normal distribution was assessed using the Kolmogorov–Smirnov-test. Categorical variables are provided with percentages. Descriptive statistics were used for demographic variables and clinical characteristics. Trauma mechanism, injury characteristics, and severity of injuries [classified with the injury severity score (ISS)] were examined. For the detection of associations between qualitative variables, a Chi-square test was performed. For the comparison between categorical and continuous variables, the Student’s *t* test or the Mann–Whitney *U* test were performed.

### Statistical evaluation of changes over the time

To detect time-dependent changes, we created a continuous numerical variable (“day of accident”) for each patient separately (*t* = 1,2,…) from January 1995 to December 2019. Linear regression analysis was used for numerical variables, and logistic regression analysis was used for categorical variables. For statistical analysis, the variable time in years (day of accident/365.25) was used. A two-sided *p* value of less than 0.05 was considered to indicate statistical significance. The Kaplan–Meier method was used to provide survival estimates, which were assessed with a log-rank test. For the mortality assessment, three time-dependent events were defined: acute-phase death (within the first 24 h after the trauma), late-phase death (after the acute phase within the hospital stay), and overall survival (death at any time after the trauma). Patients who died of unrelated causes were considered to have been censored. Univariate Cox regression analysis was performed for evaluation of potential prognostic factors on the late-phase survival. Age, severe TBI (AIS ≥ 3 points), and injury severity (ISS score) were included in the univariate Cox regression analysis as potential confounders. Only significant factors (*p* < 0.05) in the univariate analysis were entered into the multivariate analysis. Stepwise forward multivariate Cox regression analysis was performed for identification of outcome prognosticators. All statistical analyses were performed using IBM SPSS Statistics Version 26.0.

## Results

### Study population

**Fig. 1:**
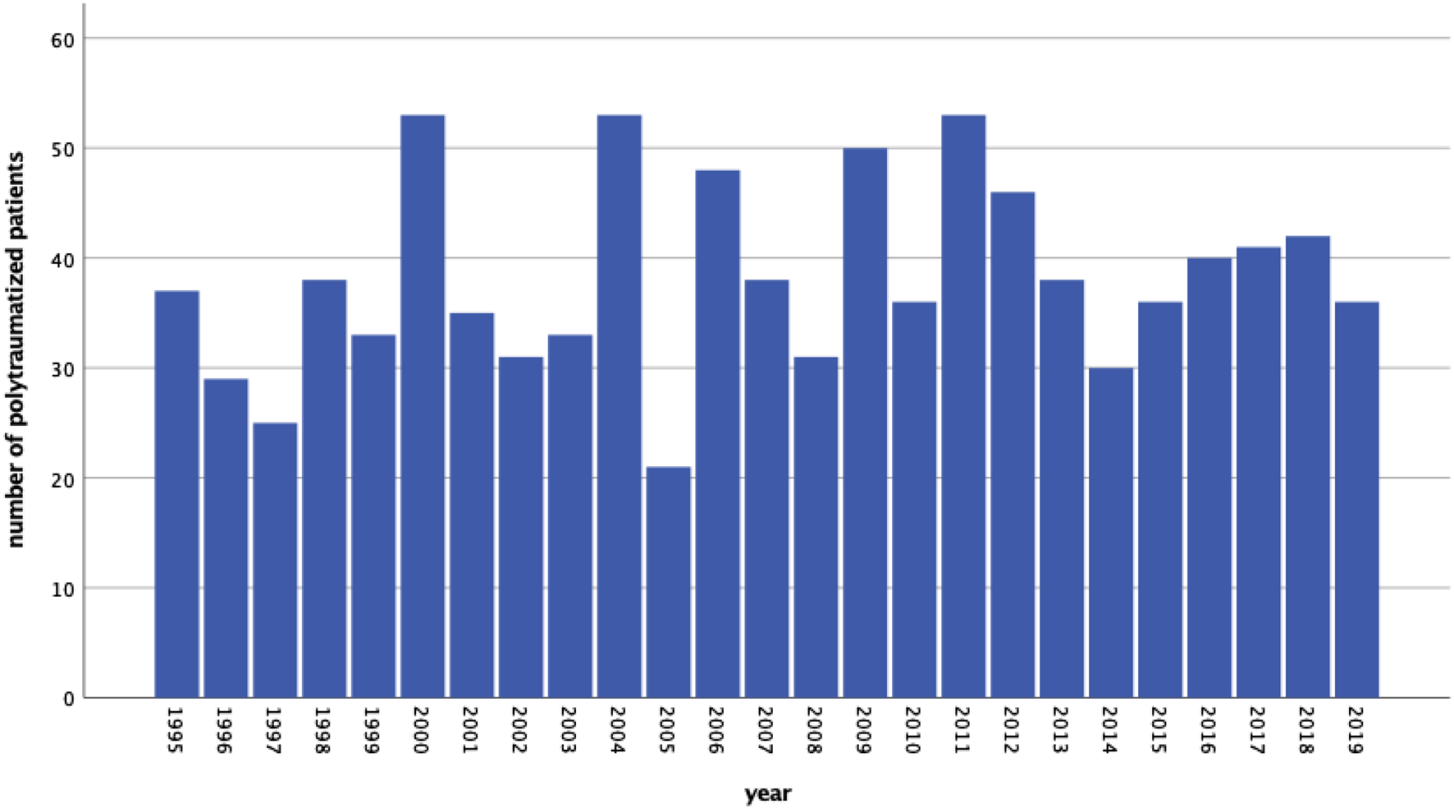
The rates of polytraumatized patients remained constant over time (linear regression: *p* = 0.291)

**Fig. 2:**
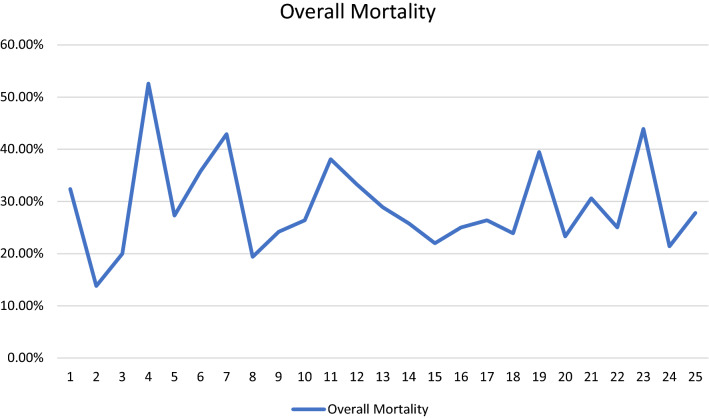
Overall mortality rates in polytraumatized patients over the years without significant changes over time

From January 1995 to December 2019, 953 adult polytraumatized patients were enrolled consecutively. The number of polytraumatized patients remained constant over time (linear regression: *p* = 0.291, Fig. 1). Clinical characteristics are listed in Table 1. Linear regression revealed an increase of age per study year by 2 years on average (*p* = 0.014). Logistic regression revealed a significant increase of the rates of geriatric (age beyond 65 years) polytraumatized patients over time (HR 1.289; 95% CI 1.020–1.630; *p* = 0.034) (Fig. 2).

**Table 1:** Clinical characteristics of 953 polytraumatized patients (ISS = Injury Severity Score)

Patients (*n* = 953)	*n*	%
*Sex*		
Female	285	29.9%
Male	668	70.1%
Age (median; IQR)	39	IQR 17–96
ISS (median; IQR)	34	IQR 18–75
*Injury mechanism*		
Traffic-related accident	561	58.9%
Fall from great height (> 3 m)	233	24.4%
Fall from lesser height	61	6.4%
Penetrating injury	32	3.4%
Other	66	6.9%
*Injury pattern*	638	66.9%
Traumatic brain injury		
Thoracic injury	756	79.3%
Abdominal injury	390	40.9%
Extremity injury	677	71.0%
*Mortality*	283	29.7%
Acute-phase death	182	64.3%
Late-phase death	98	34.7%

### Injury severity, trauma mechanism, and injury pattern

No changes of the injury severity as seen by the ISS could be detected over time (linear regression: *p* = 0.488), although the ISS decreased significantly with rising age of the patients (linear regression: *p* = 0.002). No increase in the rates of severe TBI could be seen over time or with rising age of the patients. Traumatic brain injury (TBI) was seen in 66.9% (*n* = 638) of patients, in whom a severe TBI was detected in 65.7% (*n* = 419). There was a significant increase of falls from lesser height (< 3 m height) over time (logistic regression: HR 1.550; 95% CI 1.087–2.211, *p* = 0.015) and of penetrating injuries (logistic regression: HR 1.864; 95% CI 1.133–3.066, *p* = 0.014). Additionally, a significant change of the injury patterns could be seen with rising age: a significant increase in falls from lesser height (logistic regression: HR 1.051; 95% CI 1.037–1.066, *p* < 0.001) as well as a significant decrease in falls from greater height (HR 0.987; 95% CI 0.979–0.995, *p* = 0.002) could be documented with rising age of the patients. Geriatric polytraumatized patients did not show significantly higher rates of severe TBI compared to younger ones (*p* = 0.420). Falls from lesser height were found significantly more often in the geriatric group of patients as mechanism of injury compared to younger ones (Chi-square: 20.1% vs. 3.9%, *p* < 0.001).

### Mortality and late-phase survival

**Fig. 3:**
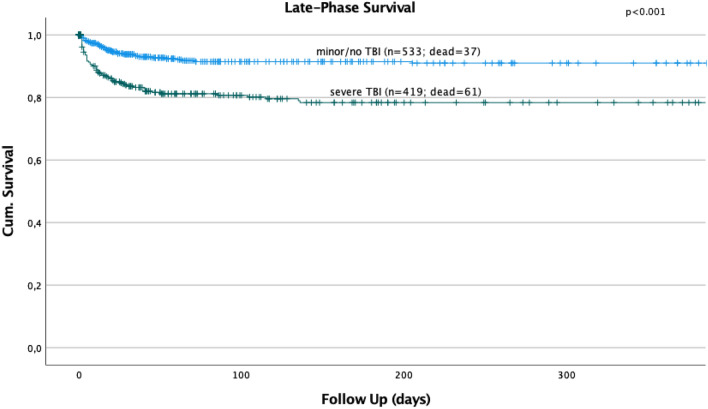
Severe TBI showed a significant influence on the late-phase survival in polytraumatized patients (*p* < 0.001)

**Fig. 4:**
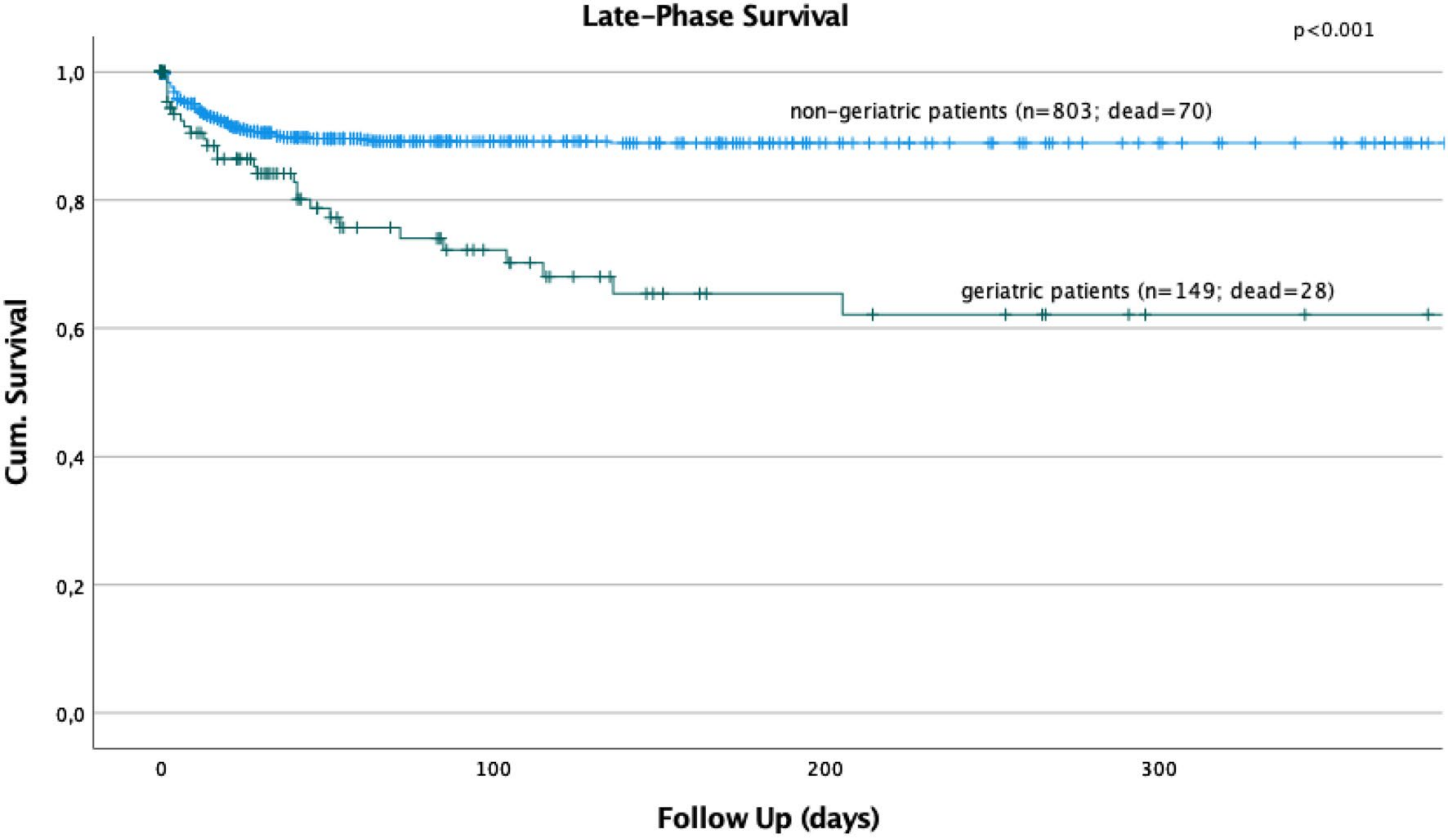
Age > 65 years of age showed a significant influence on the late-phase survival of polytraumatized patients

There was no statistically significant change of the overall, acute-phase, or late-phase mortality over time (Fig. 2). Logistic regression revealed a significant influence of age on the overall mortality (HR 1.017; 95% CI 1.010–1.025, *p* < 0.001) and on the late-phase mortality (HR 1.026; 95% CI 1.015–1.037, *p* < 0.001). The relative risk of in-hospital mortality and late-phase mortality increased with rising age of the patients by 1.7% and 2.2% per patient’s year. The injury severity had a significant influence on the overall mortality (HR 1.087; 95% CI 1.073–1.101, *p* < 0.001) and on the acute-phase mortality (HR 1.117; 95% CI 1.099–1.135, *p* < 0.001) but not on the late-phase mortality (HR 0.992; 95% CI 0.977–1.008, *p* = 0.327). Severe TBI (HR 2.737; 95% CI 1.763–4.248; *p* < 0.001) and age beyond 65 years (HR 2.507; 95% CI 1.666–3.772; *p* < 0.001) were identified as independent prognostic factors on the late-phase survival after multivariate regression analysis (Figs. 3, 4).

## Discussion

Our study contributes several new insights reporting the epidemiology and outcomes of adult polytraumatized patients within the past 25 years.

First, we were able to show a significant increase in the median age of our polytraumatized patients by 2 years in average as well as a significant increase in the rate of geriatric polytraumatized patients within the past 25 years, similar to previous reports [5, 9, 10]. In contrast to that, the injury severity stayed stable over the study period, but showed a significant decrease with rising age of the patients. Although an increase of the incidence of TBI in elderly trauma patients mainly caused by road injuries and falls has been shown [11], the rates of severe TBI remained constant in our study cohort over time.

Second, characteristic changes were seen in our study cohort regarding the injury mechanism of polytraumatized patients. In accordance with the observed changes in the injury patterns, the number of traffic-related accidents in our study cohort decreased significantly within the past 25 years as demonstrated in the previous studies [5, 7]. A significant increase in penetrating injuries (stab wounds and gun-shot wounds) could be seen over time, although penetrating injuries remain uncommon in most European countries [29, 30]. Another remarkable result of our study is a significant increase of polytrauma due to falls from lesser height over time and with rising age of the patients, especially in the geriatric cohort of our polytraumatized patients. These findings might reflect the frailty of this specific patient cohort leading to major trauma despite low-energy trauma as injury pattern.

Third, no significant change of the overall, acute-phase, or late-phase death rates over time could be seen in our study cohort, although reports from the late 90s showed a continuous reduction of mortality in multiple trauma patients [5]. Within the last years, remarkable changes in trauma care systems have been achieved including different transfusion protocols [31] and different pre-hospital treatment strategies [32]. As demonstrated in recent studies, exsanguination is still the predominant cause for acute-phase deaths of polytraumatized patients [33], whereas TBI remains the main cause of death within the late-phase of the trauma [9, 33]. We were able to demonstrate that the injury severity is a significant prognostic factor for the acute-phase survival of polytraumatized patients, whereas, in contrast to that, the rising age of our patients had a significant influence on the overall- and especially the late-phase mortality of our polytraumatized patients. With rising age, the relative risk of overall in-hospital mortality and late-phase mortality increased by 1.7% and 2.2% per patient’s year. In contrast to that, our younger polytraumatized patients tend to die more often within the acute phase. After multivariate analysis, severe TBI and age beyond 65 years were identified as independent prognostic factors in the late-phase survival of polytraumatized patients.

### Limitation

This retrospective non-randomized, single-center analysis has the characteristic limitations of registry data and post hoc analyses. There are no data on functional outcome and quality-of-life parameters available. Furthermore, long-term mortality was not evaluated in this study. Due to the retrospective design of this study, there might be an inherent selection bias. The strength of this study and sign of quality is the careful analysis of data in all consecutively included patients.

## Conclusion

The number of polytraumatized patients remained constant over the 25-year study period. Also, the mortality rates remained stable over time, although a significant increase of the average age of polytraumatized patients could be seen with stable injury severity scores. Severe TBI and age beyond 65 years remained independent prognostic factors on the late-phase survival of polytraumatized patients.

Taken together, our study demonstrates significant characteristic changes in the demographic variables and clinical characteristics of polytraumatized patients in a European level 1 trauma center over the past 25 years. The lack of improvement in mortality rates could be explained by the constant increase of the median age accompanied by a constant percentage of severe TBI over time reflecting the frail polytraumatized patient.

## Data Availability

The data used/analyzed are available from the corresponding author upon reasonable request.
